# Tetraspanin-8 promotes hepatocellular carcinoma metastasis by increasing ADAM12m expression

**DOI:** 10.18632/oncotarget.9769

**Published:** 2016-06-01

**Authors:** Tingting Fang, Jiajia Lin, Yanru Wang, Guangnan Chen, Jing Huang, Jie Chen, Yan Zhao, Ruixia Sun, Chunmin Liang, Binbin Liu

**Affiliations:** ^1^ Key Laboratory of Carcinogenesis and Cancer Invasion (Fudan University), Ministry of Education, Shanghai, P. R. China; ^2^ Laboratory of Tumor Immunology, Department of Anatomy, Histology, and Embryology, School of Basic Medical Sciences, Fudan University, Shanghai, P. R. China; ^3^ The Second Affiliated Hospital, School of Medicine, Zhejiang University, Hangzhou, P.R. China

**Keywords:** ADAM12, hepatocellular carcinoma, metastasis, TSPAN8

## Abstract

Recent evidence indicates that tetraspanin-8 (TSPAN8) promotes tumor progression and metastasis. In this study, we explored the effects of TSPAN8 and the molecular mechanisms underlying hepatocellular carcinoma (HCC) metastasis using various HCC cell lines, tissues from 149 HCC patients, and animal models of HCC progression. We showed that elevated expression of TSPAN8 promoted HCC invasion *in vitro* and metastasis *in vivo*, but did not influence HCC cell proliferation *in vitro*. Increased TSPAN8 expression in human HCC was predictive of poor survival, and multivariate analyses indicated TSPAN8 expression to be an independent predictor for both postoperative overall survival and relapse-free survival. Importantly, TSPAN8 enhanced HCC invasion and metastasis by increasing ADAM12m expression. We therefore conclude that TSPAN8 and ADAM12m may be useful therapeutic targets for the prevention of HCC progression and metastasis.

## INTRODUCTION

Hepatocellular carcinoma (HCC) is one of the most common solid tumors and the third leading cause of cancer mortality in the world [1]. It is characterized by a high probability of recurrence and metastasis [2]. Although successful curative hepatectomy has significantly improved the survival of patients with HCC, the prognosis is still poor as a consequence of tumor invasiveness, frequent intrahepatic spread, and extrahepatic metastasis. Additionally, the molecular mechanisms underlying HCC metastasis have not been elucidated.

The TSPAN8 family of integral membrane proteins is characterized by the presence of four highly conserved transmembrane domains [3]. Tetraspanin-8 (TSPAN8), also known as CO-029, TM4SF3, and D6.1A (in rats), is a member of the transmembrane 4 superfamily. Over-expression of TSPAN8 has been reported in many digestive system neoplasms including colorectal, liver, esophageal, and pancreatic cancer, and is associated with poor prognosis [4–7]. TSPAN8 also promotes the proliferation and metastasis potential of tumors by inducing angiogenesis and cell migration [7–10], but the detailed molecular mechanisms underlying the role of TSPAN8 in promoting tumor progression and metastasis are unclear.

In the present study, TSPAN8 expression was evaluated in various HCC cell lines with stepwise metastatic potential. Additionally, the expression of TSPAN8 and its relationship to patient survival was evaluated in specimens from 149 HCC patients. Up-regulation of TSPAN8 in non-metastatic human HCC cell lines increased the metastatic potential while down-regulation of TSPAN8 in highly metastatic HCC cell lines decreased the metastatic potential. These data suggested that TSPAN8 was involved in HCC metastasis. Importantly, ADAM12m, a membrane integrated disintegrin and metalloproteinase, was positively correlated with TSPAN8 expression. The results suggest that ADAM12m contributes to TSPAN8-mediated HCC metastasis.

## RESULTS

### TSPAN8 is up-regulated in HCC cell lines with higher metastatic potential and is an independent predictor for both overall survival and relapse-free survival in HCC patients

**Figure 1:**
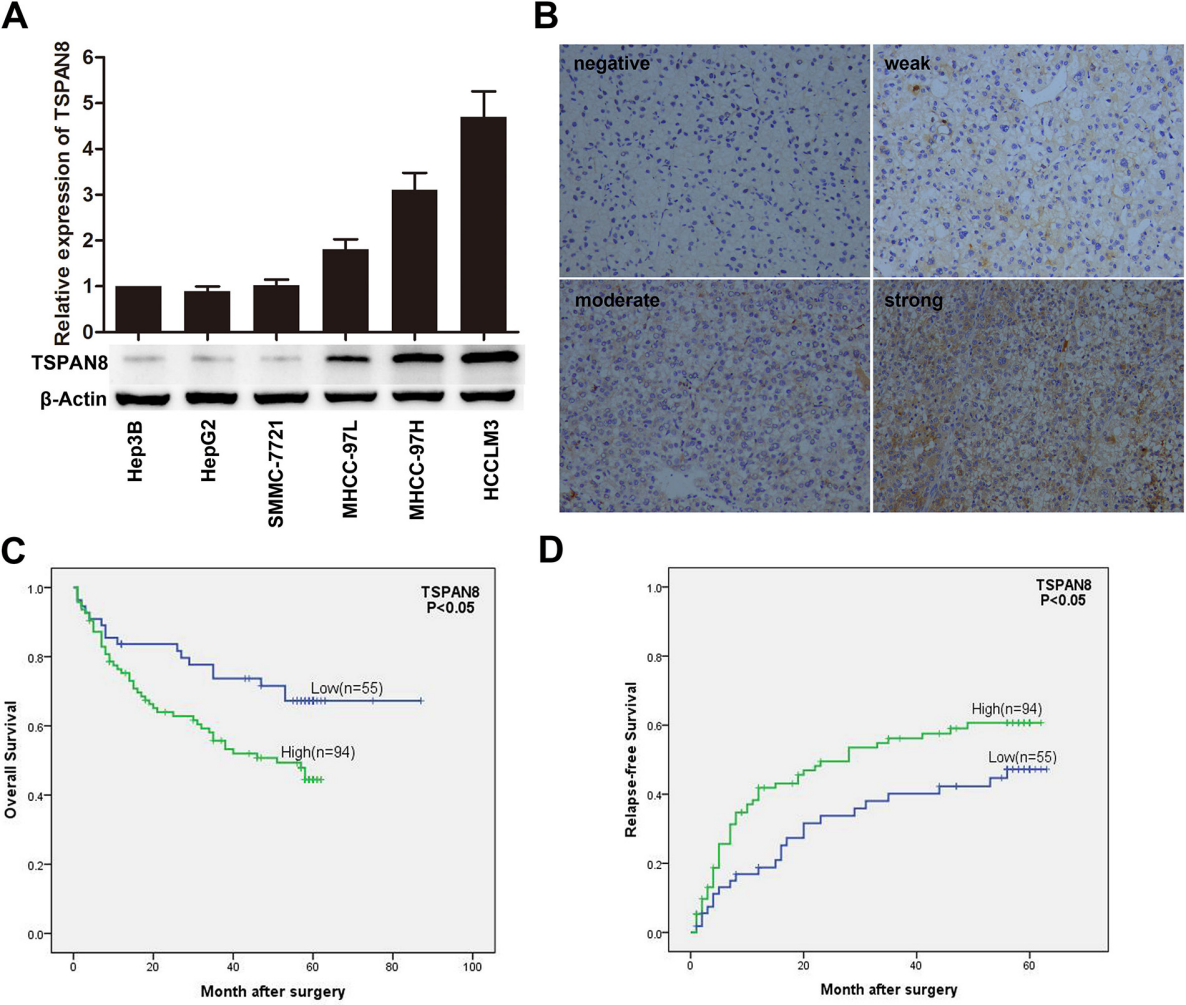
TSPAN8 is up-regulated in HCC cells **A.** TSPAN8 protein and mRNA expression in a variety of HCC cell lines with stepwise metastatic potential. Western blotting with β-actin as the loading control. RNA levels were normalized to GAPDH and the results are expressed as the mean ± SEM (n = 3) (*P < 0.05). **B.** Representative IHC images of human HCC sections stained for TSPAN8. Membranous staining is observed in the cancer cells (magnification, ×200). **C.** and **D.** Kaplan-Meier survival analysis curves for 149 HCC patients with respect to TSPAN8 expression. High TSPAN8 levels were correlated with decreased OS and RFS (*P < 0.05).

To explore the role of TSPAN8 in HCC development and metastasis, we first examined the expression of TSPAN8 in a variety of HCC cell lines with stepwise metastatic potential. TSPAN8 was highly expressed in cell lines with intermediate or high metastatic potential (MHCC97L, MHCC97H, and HCCLM3 cells) compared cell lines with low or no metastatic potential (Hep3B, HepG2, and SMMC-7721 cells) (Figure 1A). In addition, TSPAN8 expression was evaluated by immunohistochemistry (IHC) in HCC tissue samples that were isolated from 149 patients. TSPAN8 was predominantly distributed on the cell membrane, and higher expression was observed in tumor compared to peri-tumor tissue (Figure 1B). TSPAN8 expression as well as the clinical and pathologic characteristics of the 149 HCC patients who participated in the study are summarized in Table 1. The data showed that high TSPAN8 expression was significantly correlated with tumor thrombus (*P* = 0.017), differentiation (*P* = 0.037), and TNM stage (*P* = 0.036). Multivariate analyses revealed that TSPAN8 expression was an independent predictor for overall survival (OS) and relapse-free survival (RFS) (Table 2). Furthermore, 63.1% of patients had high TSPAN8 expression. Kaplan-Meier survival analysis indicated that high expression of TSPAN8 was significantly correlated with decreased OS and RFS (Figure 1C & 1D; *P* < 0.05). These results indicated that TSPAN8 expression was positively correlated with the invasive and metastatic potential of HCC cells, which was consistent with previous studies [4].

**Table 1:** Correlation between TSPAN8 expression and clinicopathologic in HCC patients

Variables		Patients		TSPAN8 staining		*P* value
		Number	%	low	high	
Gender	Male	126	84.6	46	80	0.811
	Female	23	15.4	9	14	
Age(Year)	≤50	68	45.6	23	44	0.555
	>50	81	54.4	32	50	
HBsAg	Positive	125	84.0	45	79	0.726
	Negative	24	16.0	10	15	
HCVAb	Positive	6	4.0	3	3	0.498
	Negative	143	96.0	52	91	
AFP (ng/ml)	≤20	62	41.6	23	39	0.969
	>20	87	58.4	32	55	
ALT (U/L)	≤75	142	95.3	53	89	0.639
	>75	7	4.7	2	5	
Tumor size (cm)	≤5	65	43.6	27	38	0.303
	>5	84	56.4	28	56	
Tumor number	Single	146	98.0	46	80	0.811
	Multiple	23	2.0	9	14	
Tumor encapsulation	Present	67	45.0	20	47	0.106
	Absent	82	55.0	35	47	
Tumor differentiation	I/II	115	77.2	46	64	0.037*
	III/IV	34	22.8	9	30	
Tumor thrombus	Absent	90	60.4	38	46	0.017*
	Present	59	39.6	17	48	
TNM stage	I	70	47.0	32	38	0.036*
	II/III	79	53.0	23	56	

Chi-square test was used in all analysis.

*HBsAg* hepatitis B surface antigen, *AFP* alpha-fetoprotein, *TNM* tumor-node-metastasis.

* P<0.05.

**Table 2:** Univariate and multivariate analyses of factors associated with survival and recurrence

Variables	Overall survival			Relapse-free survival		
	Univariate *P* value	Multivariate		Univariate *P* value	Multivariate	
		HR (95 % CI)	*P* value		HR (95 % CI)	*P* value
Sex (female vs. male)	0.222	NA		0.128	NA	
Age (years) (≤50 vs.>50)	0.486	NA		0.165	NA	
HBsAg (positive vs. negative)	0.996	NA		0.352	NA	
HCVAb (positive vs. negative)	0.813	NA		0.371	NA	
Serum AFP, ng/mL (≤20 vs.>20)	0.134	NA		0.050	2.18(1.34-3.36)	0.002**
Serum ALT, U/L (≤75 vs.>75)	0.876	NA		0.906	NA	
Tumor size (diameter, cm) (≤5 vs.>5)	<0.001**	1.82(1.17-3.71)	0.013*	<0.001**	NS	
Tumor number (multiple vs. single)	0.112	NA		0.169	NA	
Tumor thrombus (absent vs. present)	<0.001**	2.08(1.41-4.05)	0.001**	0.003**	0.51(0.32-0.82)	0.006**
Tumor encapsulation (absent vs. present)	0.034*	NA		0.010**	NA	
TNM stage (II/III vs. I)	<0.001**	NS		0.013*	NA	
Tumor differentiation (III/IV vs I/II.)	0.269	NA		0.060	NA	
TSPAN8 expression (high vs. low)	0.016*	2.39(1.05-3.18)	0.034*	0.050	1.78(1.08-2.93)	0.025*

NA, not adopted; NS, not significant; AFP, alpha-fetoprotein; HBsAg, hepatitis B surface antigen; 95%CI, 95% confidence interval; Cox proportional hazards regression model.

* P<0.05

** P<0.01.

### TSPAN8 promotes the invasion and migration of HCC cells but not proliferation *in vitro*

**Figure 2:**
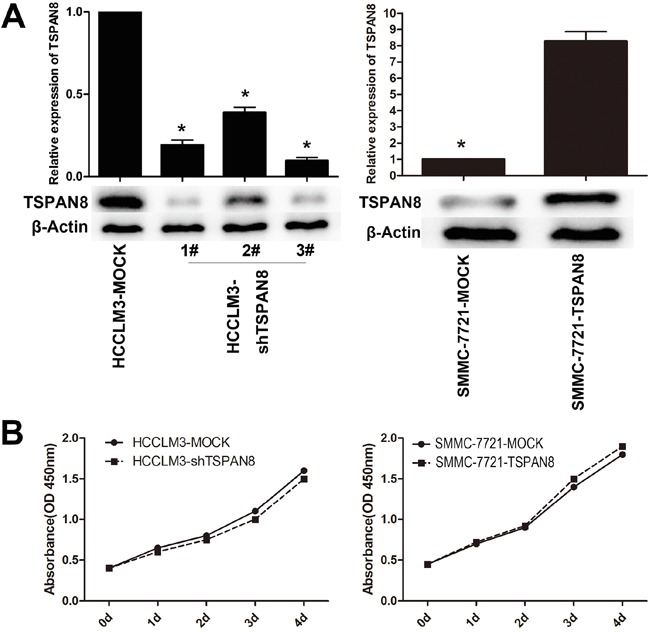
Modulation of TSPAN8 expression had no effect on the proliferation of HCC cells *in vitro* **A.** Quantitative RT-PCR and western blotting analysis of the TSPAN8 expression in HCCLM3 and SMMC-7721 cells, which was modified by shRNA and cDNA transfection. **B.** Cell count kit-8 (CCK-8) HCC cell proliferation assays. Down-regulation of TSPAN8 in HCCLM3 cells by shRNA had no significant effect on cell proliferation at any of the indicated times (*P* > 0.05).

The effects of TSPAN8 on the biological behavior of HCC cells were analyzed in two HCC cell lines: HCCLM3 cells with higher TSPAN8 expression and SMMC-7721 cells with lower TSPAN8 expression. TSPAN8 expression in HCCLM3 cells was down-regulated by shRNA using a recombinant lentiviral vector (pGCSIL-GFP-shRNA-TSPAN8). We also over-expressed TSPAN8 in SMMC-7721 cells using a recombinant lentiviral vector (pGC-FU-GFP-TSPAN8). Knock-down or over-expression of TSPAN8 in HCC cell lines was successfully detected by quantitative real-time PCR (qRT-PCR) and western blot (Figure 2A; *P* < 0.05). We also confirmed that down-regulation of TSPAN8 in HCCLM3 cells had no significant effect on cell proliferation at any of the indicated times. Similarly, over-expression of TSPAN8 did not affect SMCC-7721 cell proliferation (Figure 2B; *P* > 0.05).

**Figure 3:**
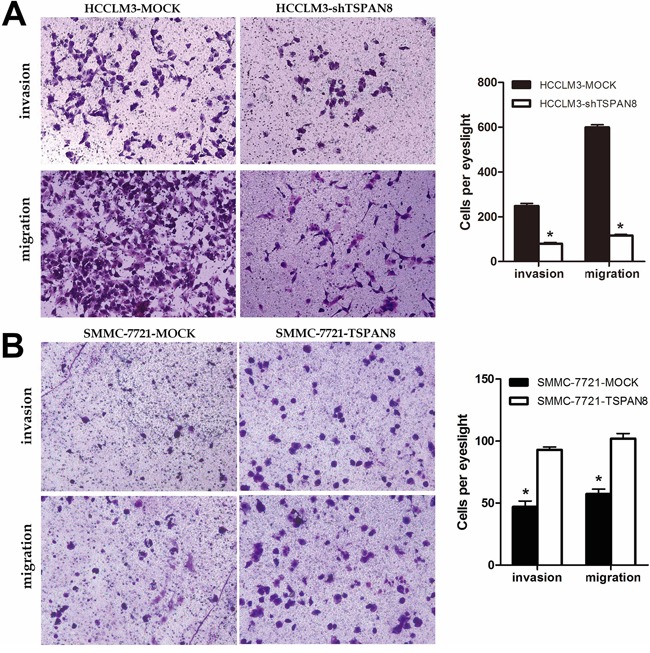
High expression of TSPAN8 promoted HCC metastasis and invasion *in vitro* **A.** and **B.** The invasion and migration of cancer cells was measured using transwell assays. After TSPAN8 knockdown, HCCLM3 transfected with shRNA exhibited a significant reduction in the number of invaded cells compared to HCCLM3-MOCK cells (*P < 0.05), while SMMC-7721 cells with up-regulation of TSPAN8 had a significantly higher number of invaded cells compared to the negative controls (*P < 0.05).

We next analyzed HCC cell migration and invasion using transwell assays. HCCLM3-shTSPAN8 cells exhibited a significant decrease in the number of invaded cells compared to HCCLM3-MOCK cells (Figure 3A; *P* < 0.05), while the SMMC-7721-TSPAN8 cells had a significantly higher number of invaded cells compared to the negative controls (Figure 3B; *P* < 0.05). Similar results were obtained in migration assays (Figure 3; *P* < 0.05). These results suggested that TSPAN8 promoted HCC cell invasion and migration but did not affect proliferation.

### TSPAN8 promotes HCC growth and metastasis in tumor xenograft models

**Figure 4:**
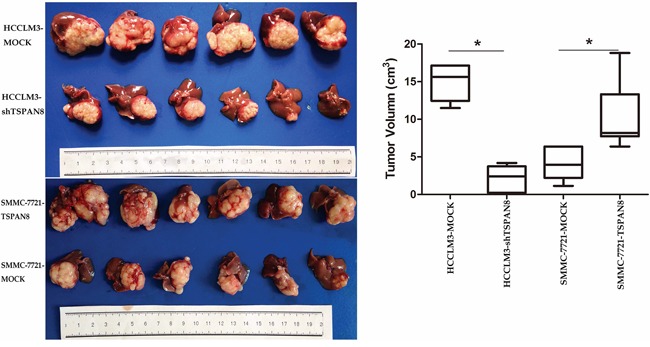
High expression of TSPAN8 promoted HCC growth *in vivo* The volume of the tumors derived from HCC isogenic cell lines was measured *in vivo* for 7 weeks. TSPAN8 knockdown in the HCCLM3-shTSPAN8 group resulted in a significant decrease in tumor size compared to the control group, while TSPAN8 over-expression in the SMMC-7721-TSPAN8 group resulted in a significant increase in tumor size (*P < 0.05).

To further investigate the role of TSPAN8 in HCC, we generated orthotropic HCC mouse models. TSPAN8 knock-down in the HCCLM3-shTSPAN8 group resulted in a significant decrease in tumor size compared to controls, while TSPAN8 over-expression in the SMMC-7721-TSPAN8 group resulted in a significant increase in tumor size (Figure 4; *P* < 0.05).

**Figure 5:**
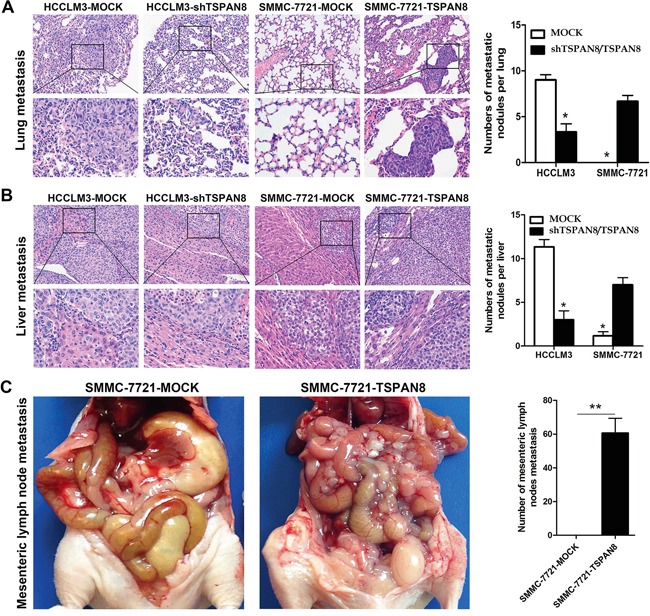
High expression of TSPAN8 promoted HCC metastasis and invasion *in vivo* **A.** Representative picture of lung metastases and a comparison of lung metastatic nodule number between different levels of TSPAN8 in the HCCLM3 or SMMC-7721 groups (magnification, ×200 and ×400) (*P < 0.05). **B.** Representative picture of intrahepatic metastases and a comparison of intrahepatic metastatic nodule number between different levels of TSPAN8 in the HCCLM3 or SMMC-7721 groups (magnification, ×200 and ×400). The HCCLM3-MOCK and SMMC-7721-TSPAN8 groups had significant lung and intrahepatic metastases, while the HCCLM3-shTSPAN8 and SMMC-7721-MOCK groups had less lung and liver metastases (*P < 0.05). **C.** Over-expression of TSPAN8 in SMMC-7721 promoted spontaneous mesenteric lymph node metastasis (**P < 0.01). Metastatic nodules were counted manually and the number of metastases per mouse was presented as the mean ± SD. The *P* values were determined by Student's t-tests.

The HCCLM3-MOCK and SMMC-7721-TSPAN8 groups exhibited lung and intrahepatic metastasis, while the HCCLM3-shTSPAN8 and SMMC-7721-MOCK groups had less metastasis to these sites. The number of lung and intrahepatic metastatic nodules revealed by hematoxylin and eosin staining was significantly higher in the SMMC-7721-TSPAN8 than in the SMMC-7721-MOCK group (Figure 5A & 5B; *P* < 0.05). In addition, over-expression of TSPAN8 in the SMMC-7721 group promoted spontaneous mesenteric lymph node metastasis, while the control group had almost no metastasis to mesenteric lymph nodes (Figure 5C; *P* < 0.01).

### ADAM12m expression is positively correlated with TSPAN8 expression

Previous studies have described the roles matrix degrading metalloproteinases in cancer [11], including the roles of matrix metalloproteinases (MMPs), which have been associated with a variety of human malignancies [12]. For example, the roles of ADAMs in cancer are currently under investigation [13]. The expression of MMP-2, MMP-9, and ADAM12m has been shown to have a crucial role in cancer cell invasion and metastasis through promoting degradation of numerous peri-cellular substrates [14, 15]. Our *in vitro* studies demonstrated that up-regulation of TSPAN8 expression promotes HCC cell migration and invasion. We then aimed to determine whether TSPAN8-enhanced HCC cell invasion and metastasis were associated with a matrix degradation enzyme.

**Figure 6:**
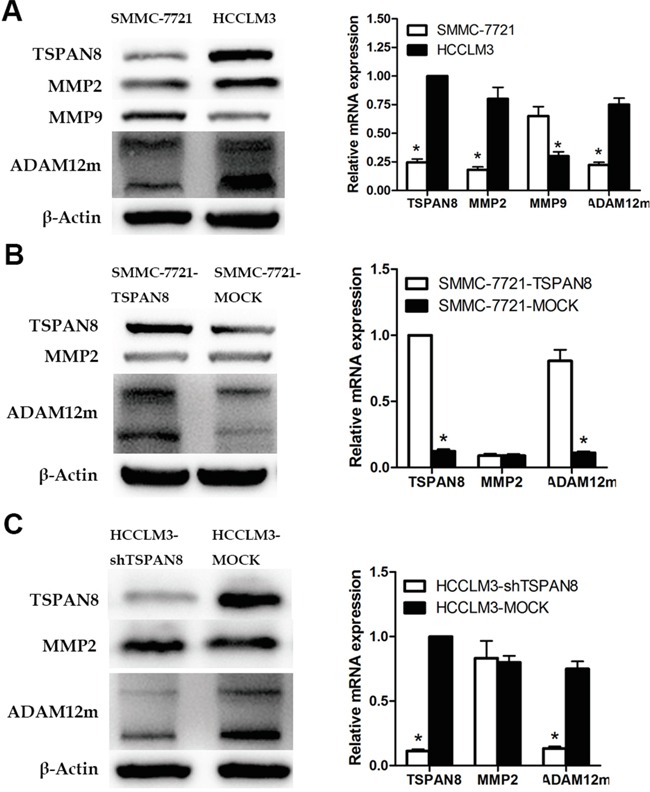
TSPAN8 up-regulated ADAM12m expression **A.** Western blotting and qRT-PCR showing MMP2, MMP9, and ADAM12m protein and mRNA expression in HCCLM3 and SMMC-7721 cells (*P < 0.05). **B.** Western blotting and qRT-PCR showing MMP2 and ADAM12m protein and mRNA expression in SMMC-7721-TSPAN8 and SMMC-7721-MOCK cells (*P < 0.05). **C.** Western blots and qRT-PCR showing MMP2 and ADAM12m protein and mRNA expression in HCCLM3-shTSPAN8 and HCCLM3-MOCK cells.

We first examined the expression of MMP-2, MMP-9, and ADAM12m in SMMC-7721 (low metastatic potential) and HCCLM3 cells (high metastatic potential). Intriguingly, western blotting revealed that the expression of MMP-2 and ADAM12m was higher in HCCLM3 compared to SMCC-7721 cells, which was consistent with the expression of TSPAN8. However, the expression of MMP-9 was increased in SMMC-7721 compared to HCCLM3 cells (Figure 6A; *P* < 0.05). We confirmed the regulation of MMP-2 and ADAM12m by TSPAN8 through up- or down-regulation of TSPAN8 expression in SMMC-7721 or HCCLM3 cells. The expression of ADAM12m in HCCLM3-shRNA cells was assayed by quantitative real-time RT-PCR and western blotting. Interestingly, ADAM12m was decreased by 86.2% in these cells compared to HCCLM3-MOCK cells. In SMMC-7721-TSPAN8 cells, the expression of ADAM12m was increased by more than four-fold compared to SMMC-7721-MOCK cells (Figure 6B & 6C). At the same time, MMP-2 expression did not change in response to TSPAN8 knock-down or over-expression (Figure 6B & 6C; *P* >0.05). Our data demonstrated that ADAM12m expression was positively correlated with TSPAN8 expression in HCC cells.

### TSPAN8 induced invasion of HCC cells by increasing ADAM12m expression

**Figure 7:**
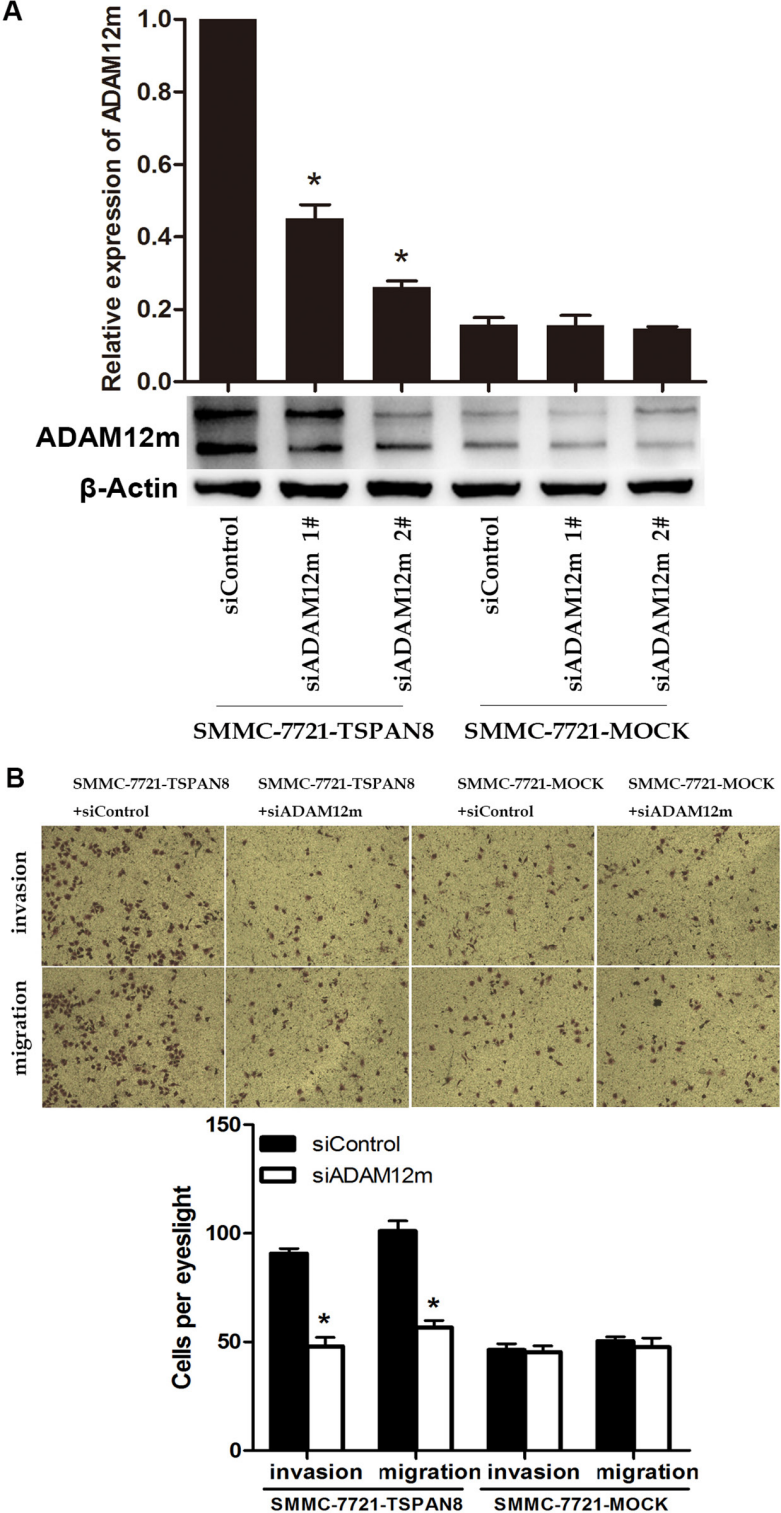
TSPAN8 induced HCC invasion and motility by up-regulating ADAM12m expression **A.** Western blots and qRT-PCR analysis of ADAM12m expression in SMMC-7721 cells in which ADAM12m was modulated by siRNA or a a control siRNA (*P < 0.05). **B.** Transwell migration and invasion assays of SMMC-7721-TSPAN8 and SMMC-7721-MOCK cells treated with ADAM12m siRNA or a control oligonucleotide. Down-regulation of ADAM12m expression decreased TSPAN8-induced invasion and migration to that of cell lines that lacked exogenous expression of TSPAN8 (*P < 0.05).

To explore the mechanisms underlying the promotion of HCC cell invasiveness by TSPAN8, transwell assays were performed to identify ADAM12m-associated factors. The efficiency of ADAM12m knock-down in SMMC-7721 cells was confirmed by western blotting and qRT-PCR (Figure 7A). We chose a more effective siRNA (#2) in the following assays. Using transwell assays, we examined the effects of ADAM12m on invasion and migration, which showed that down-regulation of ADAM12m expression decreased TSPAN8-induced invasion and migration to the levels observed for cell lines that lacked exogenous expression of TSPAN8 (Figure 7B; *P* < 0.05). These results indicated that TSPAN8 promoted the invasion and migration of HCC cells by increasing ADAM12m expression.

## DISCUSSION

We demonstrated that TSPAN8 promotes HCC metastasis by increasing ADAM12m expression. First, we determined that TSPAN8 expression was lower in noninvasive HCC cells and higher in HCCLM3 cells that had the highest metastatic potential. Second, we confirmed that TSPAN8 expression was markedly increased in HCC tissue compared to adjacent non-tumorous tissues. Finally, over-expression of TSPAN8 contributed to HCC growth and metastasis *in vivo* and was associated with a poor prognosis in HCC patients.

TSPAN8 belongs to the tetraspanin family of integral membrane proteins, which is characterized by the presence of four highly conserved transmembrane domains [3]. Although the tumor growth-promoting and metastasis-promoting activities of TSPAN8 may be attributed to its capacity to induce tumor angiogenesis and promote cancer cell proliferation, migration, and invasion [7–10], our results demonstrated that TSPAN8 contributed to the migration and invasion of HCCs but not proliferation *in vitro*. These data indicated that the ability of TSPAN8 to promote HCC growth might depend on interactions with host cells.

Previous studies have demonstrated that tetraspanins can regulate cell proliferation, cell-cell and cell-matrix interactions, and cell motility through interaction with many transmembrane, cytosolic, and other signaling and/or adhesive proteins [7–10]. Some members of the tetraspanin family such as CD9 and CD82 are believed to function as metastasis suppressors [20, 21]. However, others members such as TSPAN8 promote metastasis [22, 23]. TSPAN8 over-expression has been observed in gastrointestinal cancer, pancreatic cancer, colorectal cancer liver metastases, and intrahepatic spread of HCC [5, 7, 9, 24, 25]. Our observations are consistent with these findings in that over-expression of TSPAN8 in SMMC-7721 HCC cells (low metastatic potential) led to higher intrahepatic metastatic ability, whereas knock-down of TSPAN8 in HCCLM3 cells (high metastatic potential) prevented intrahepatic metastasis. We confirmed that over-expression of TSPAN8 resulted in an increase in tumor size and mesenteric lymph node metastasis in animal models of HCC. At the same time, we did not observe any direct effect of TSPAN8 on the proliferation of HCC cells *in vitro*, which was consistent with previous observations [24]. Of note, our studies of HCC patients indicated that TSPAN8 expression was not correlated with tumor size. This is in contrast to the expectation that TSPAN8 would promote tumor growth, which was suggested by studies using nude mouse models of HCC. This may be explained by heterogeneity among different types of HCC or by differences in the tumor microenvironment between humans and nude mice. The results suggested that TSPAN8 might contribute to HCC metastasis through other mechanisms besides cell growth.

The metastatic process includes multiple events such as cell invasion, migration, and degradation of extracellular matrix (ECM). MMPs and ADAMs contribute to this process. Therefore, modulating the biosynthesis and activation of associated proteins such as MMPs and ADAMs could influence tumor cell invasion [24]. MMP-2 and MMP-9 are particularly important because they play pivotal roles in the degradation of ECM to promote metastasis [14, 27]. Our study of human HCC cells suggested that TSPAN8 might enhance invasion and migration through up-regulation of MMP-2 and MMP-9. However, we found that neither down-regulation nor up-regulation of TSPAN8 altered the expression of MMP-2 and MMP-9 in HCC cells.

The ADAMs are a family of multi-domain glycoproteins that are highly homologous to the class III snake venom metalloprotease-disintegrins [28], and half of the ADAMs are predicted to be active metalloproteinases. Furthermore, ADAMs are characterized by putative integrin ligand properties through the disintegrin domain. Recent studies have demonstrated expression of several ADAMs in cell lines derived from human tumors [29, 30]. Over-expression of ADAM12 was also observed in several human carcinomas [31]. Human ADAM12 exists in two forms that arise from alternate splicing: the prototype membrane-anchored protein (ADAM12m) and a shorter secreted form (ADAM12s) [32]. A previous report indicated that the expression of ADAM12 was associated with tumor aggressiveness and progression in HCC [15]. Here, we demonstrated a related phenomenon, which is that the effect of ADAM12m on the progression and metastasis of HCC was associated with TSPAN8.

In conclusion, TSPAN8 is up-regulated in HCC cells with high metastatic potential and is an independent prognostic factor for RFS and OS. Furthermore, it is TSPAN8 could promote HCC migration and invasion *in vitro* by increasing ADAM12m expression. These results suggest that TSPAN8 and ADAM12m is a potential therapeutic targets for the treatment of HCC.

## MATERIALS AND METHODS

### Patients and follow-up

The HCC samples that were used to construct the tissue microarray (n = 149) were obtained from patients who had undergone curative hepatectomy and were diagnosed with abdominal lymph node metastases (LNMs) during follow-up care at Zhongshan Hospital between January 2008 and November 2009. Patients were followed until May 2013 and the longest follow-up was up to 87 months. OS was defined as the interval between surgery and either death or the last follow-up. RFS was defined as the interval between surgery and either the first recurrence or the last follow-up for patients who did not relapse. The data was censored at the last follow-up for patients who did not relapse or die. This study was approved by the Research Ethics Committee of Zhongshan Hospital (Shanghai, PR China), and written informed consent was obtained from each patient.

### Cell culture

Six human HCC cell lines were used in this study. The human HCC cell lines (Hep3B, HepG2, and SMMC-7721) were obtained from the Cell Bank of Shanghai Institutes of Biological Sciences, Chinese Academy of Sciences, and the stepwise metastatic HCC cell lines (MHCC97L, MHCC97H, and HCCLM3) were established in the Liver Cancer Institute at Fudan University. Cells were cultured in DMEM or MEM with high glucose (Gibco BRL, USA) supplemented with 10% fetal bovine serum (Gibco BRL, USA). All cells were incubated at 37°C in a humidified atmosphere containing 5% CO2.

### Quantitative real-time PCR

RNA was extracted from cell lines using the Trizol reagent (Invitrogen, USA) according to the manufacturer's protocol and was reverse transcribed using the PrimeScript^®^ RT Master Mix (Takara, Japan). SYBR Green fluorescence-based qRT-PCR was performed according to the manufacturer's instructions (Takara, Japan). The primers used in our study are listed in Supplementary Table 1. Relative mRNA expression levels were calculated using the –ΔCt method and expressed as 2^ (−ΔCt) based on the threshold cycle (Ct) values and were normalized to the internal control (GAPDH).

### Western blotting

Cells were treated with lysis buffer (Beyotime Institute of Biotechnology, China) for 45 min on ice. ADAM12 enrichment from HCC cell lysates was performed for glycoproteins by binding to concanavalin A agarose (Sigma Aldrich; 25 mL resin/500 mL lysate). Equal amounts of protein were separated by 10% SDS-PAGE and electrophoretically transferred to PVDF membranes (Millipore, USA) at 90–100 mA for 1.5–2 h. Membranes were blocked with PBS-0.05% Tween 20 containing 5% nonfat dry milk for 1 h and incubated with primary antibody overnight at 4°C. Membranes were then washed three times with PBS-0.05% Tween 20 and incubated with secondary antibody for 2 h at room temperature. Blots were developed using an ECL reagents (Beyotime Institute of Biotechnology, China). Each experiment was repeated at least three times. Antibodies used in the experiments included rabbit anti-human TSPAN8 antibody (Abcam, USA), rabbit anti-human MMP-2 antibody (BioWorld, USA), and rabbit anti-human MMP-9 antibody (BioWorld, USA) followed by a 1:1000 dilution of HRP-conjugated goat anti-rabbit IgG F(ab')2 antibody (Beyotime Institute of Biotechnology, China), as well as rabbit anti-human ADAM12m antibody (Sigma-Aldrich, USA) followed by a 1:1000 dilution of HRP-conjugated goat anti-rabbit IgG F(ab')2 antibody (Beyotime Institute of Biotechnology, China).

### Immunohistochemistry

IHC of paraffin sections was performed using a two-step protocol (Novolink Polymer Detection System, Novocastra, UK) according to the manufacturer's instructions. Briefly, paraffin sections were deparaffinized and the endogenous peroxidase neutralized with 0.3% H_2_O_2_. After antigen retrieval was performed in a microwave oven in pH 6.0 citrate buffer, the tissues were incubated with the primary and secondary antibodies. The tissue microarray was then stained with diaminobenzidine (DAB) and counterstained with hematoxylin. The primary antibody used was rabbit anti-human TSPAN8 (Abcam, USA).

The final scores were calculated by multiplying the staining intensity score by the percentage of positive cells. Specifically, the staining intensity scores were divided into four grades: 0 (negative), 1 (weak), 2 (moderate), or 3 (strong). The scores for the percentage of positive cells were also divided into four grades: 0, no positive cells; 1, 1–10% positive cells; 2, 11–50% positive cells; and 3, > 50% positive cells. TSPAN8 expression was defined as high if the final score was between 4–9 and it was negative if the score was between 0–3.

### Cell transfection and TSPAN8 RNAi

A TSPAN8-RNAi lentiviral vector (pGCSIL-GFP-shRNA-TSPAN8) was constructed to silence TSPAN8 expression in both *in vitro* and *in vivo* experiments. Three putative candidate sequences and one control sequence for the TSPAN8 siRNA were designed by Sigma-Aldrich as shown in Supplementary Table 2. Two putative candidate sequences for the ADAM12m siRNA were described previously [33] and are also included in Supplementary Table 2. Transfection of the siRNAs was performed using Lipofectamine 2000 (Invitrogen, USA) according to the manufacturer's instructions. The pGCSIL-GFP-shRNA-TSPAN8 and pGC-FU-GFP-TSPAN8 lentiviral vectors were constructed by Shanghai GeneChem. Using the manufacturer's protocol, pGC-FU-GFP-TSPAN8 was transfected into SMMC-7721 cells that had lower TSPAN8 expression (referred to as SMMC-7721-TSPAN8) and pGCSIL-GFP-shRNA-TSPAN8 was transfected into HCCLM3 cells that had higher TSPAN8 expression (referred to as HCCLM3-shTSPAN8). The pGCSIL-GFP and pGC-FU-GFP lentiviral vectors were used as negative controls (referred to as SMMC-7721-MOCK and HCCLM3-MOCK, respectively). TSPAN8 expression in stably transfected clones was validated by qRT-PCR and western blotting.

### Cell proliferation assays

Cells were plated at a density of 2,000 cells/well in triplicate in 96-well culture plates. At the indicated time points, 10 μL of CCK-8 solution (Dojindo, Japan) was added and after incubation for another 2 h, the OD value at 450 nm was measured using an Infinite 200 (Tecan, Switzerland). All experiments were performed three times.

### Migration and invasion assays

Cell migration assays were performed in 24-well transwells with an 8.0 μm pore polycarbonate membrane insert (Corning, USA). In total, 1 × 10^5^ cells were suspended in 100 μL DMEM with high glucose (Gibco BRL, USA) with 1% FBS (Gibo BRL, USA) and added to the upper chamber. The lower chamber was filled with 600 μL DMEM with high glucose (Gibco BRL, USA) supplemented with 10% FBS (Gibco BRL, USA). After incubation for 24 h, the cells on the upper surface of the membrane were removed and the migrated cells on the lower surface were fixed in 4% paraformaldehyde, stained with 0.1% crystal violet for 15 min at room temperature, and counted (10 fields) under a ×100 objective. The mean ± standard deviation (SD) was then calculated. Invasion assays were performed in a similar manner except that the cells were seeded onto Matrigel-coated filters (BD Biosciences, USA).

### Tumor models

Male athymic BALB/c nude mice (6–8 weeks old, Shanghai Institute of Material Medicine, Chinese Academy of Science, Shanghai, China) were manipulated and housed according to the protocols approved by the Shanghai Medical Experimental Animal Care Commission. We suspended 5 × 10^6^ HCCLM3-MOCK, HCCLM3-shRNA, SMMC-7721-MOCK, or SMMC-7721-TSPAN8 cells in 100 μL of serum-free DMEM and injected the cell suspensions subcutaneously into the flank of each mouse. The subcutaneous tumors were harvested 4 weeks after injection and cut into 1.0 mm^3^ pieces. One piece was then implanted into the left lobe of the liver of a nude mouse (n = 6 in each group). Transplanted mice were sacrificed 6 weeks later based on previous studies [34, 35], the weight and volume of transplanted tumors calculated, and the intrahepatic and lung metastases detected by microscopy.

### Statistical analyses

Statistical analyses were performed using SPSS 19.0 for Windows. Comparisons between groups were performed using Student's t-tests, Pearson χ2 tests, or Fisher's exact tests. Kaplan-Meier analyses and log-rank tests were used to perform survival analyses. A two-sided *P* < 0.05 was considered statistically significant.

## SUPPLEMENTARY TABLES


