# Supplementary material for: Tetraspanin-8 promotes hepatocellular carcinoma metastasis by increasing ADAM12m expression
Source: Oncotarget. 2016 Jun 1;7(26):40630–43. doi: 10.18632/oncotarget.9769 (PMC5130032; doi:10.18632/oncotarget.9769)
Supplement: Supplementary file 1 [file oncotarget-07-40630-s001.pdf]

Tetraspanin-8 promotes hepatocellular carcinoma metastasis by increasing ADAM12m expression

SUPPLEMENTARY TABLES

Supplementary Table S1: Primer sequences for qRT-PCR

Gene name	sequence
TSPAN8	F: 5'CATCTCTCATTGACTTATCTGGTAGC3'<br>R: 5'ACGTCCCCCTAAGGTTTGGT3'
MMP2	F: 5'AAGTCTGAAGAGCGTGAAGTTTGGA3'<br>R: 5'TGAGGGTTGGTGGGATTGGAG3'
MMP9	F: 5'AGTCCACCCTTGTGCTCTTCCC3'<br>R: 5'TCTGCCACCCGAGTGTAACCAT3'
ADAM12m	F: 5'CACCATTGAAAAACTAAGGTGTGTG3'<br>R: 5'GAGCCTGACAGGGTTGGAAG3'
**GAPDH**	F: 5'TGACTTCAACAGCGACACCCA3'<br>R: 5'CACCCTGTTGCTGTAGCCAAA3'

Supplementary Table S2: Sequences for siRNA

Sequences for TSPAN8 siRNA	
**Name**	**sequence**
Sequence-1#	F: 5'GUAUCUUGAUCCUAGCAUUdTdT3'
	R: 5'AAUGCUAGGAUCAAGAUACdTdT3'
Sequence-2#	F: 5'GUCUGAUCGCAUUGUGAAUdTdT3'
	R: 5'AUUCACAAUGCGAUCAGACdTdT3'
Sequence-3#	F: 5'GAGUUUAAAUGCUGCGGUUdTdT3'
	R: 5'AACCGCAGCAUUUAAACUCdTdT3'
Control sequence	F: 5'UUCUCCGAACGUGUCACGUTT3'
	R: 5'ACGUGACACGUUCGGAGAATT3'
**Sequences for ADAM12m siRNA**	
Sequence-1#	F: 5'CCUCGCUGCAAAGAAUGUGdTdT3'
	R: 5'CACAUUCUUUGCAGCGAGGdTdT3'
Sequence-2#	F: 5'GACCUGAUACGACUGCUGdTdT3'
	R: 5'CAGCAGUCGUAUCAAGGUCdTdT3'
